# Elevated serum microRNA 483-5p levels may predict patients at risk of post-operative atrial fibrillation

**DOI:** 10.1093/ejcts/ezw245

**Published:** 2016-07-15

**Authors:** Leanne Harling, Jonathan Lambert, Hutan Ashrafian, Ara Darzi, Nigel J. Gooderham, Thanos Athanasiou

**Affiliations:** a National Heart & Lung Institute, Hammersmith Hospital, Imperial College London, London, UK; b Department of Surgery and Cancer, St Mary's Hospital, Imperial College London, London, UK; c Department of Biomolecular Medicine, South Kensington Campus, Imperial College London, London, UK; d Institute for Child Health, University College London, London, UK

**Keywords:** Post-operative atrial fibrillation, Surgery, microRNA, Biomarker

## Abstract

**OBJECTIVES:**

Post-operative atrial fibrillation (POAF) is the commonest post-operative cardiac arrhythmia, affecting ∼1 in 3 patients undergoing coronary artery bypass grafting (CABG). Although its aetiology is complex, atrial substrate changes may pre-dispose to its onset. This study aims to ascertain the atrial microRNA signature of POAF and determine the potential for circulating microRNA as a pre-operative biomarker for this arrhythmia.

**METHODS:**

Thirty-four patients undergoing non-emergent, on-pump CABG were prospectively recruited. Right atrial biopsies were taken intra-operatively and snap frozen for RNA extraction. Plasma was obtained at 24 h pre-operatively and at 2 and 4 days post-operatively. POAF was defined by continuous Holter recording. Inter-group comparisons were performed using Student's *t*-test or analysis of variance as required. Receiver operating characteristic (ROC) analysis was used to determine the diagnostic accuracy of pre-operative serum miRNA as a POAF biomarker.

**RESULTS:**

Sixteen microRNAs were differentially expressed in the atrial myocardium of POAF patients when compared with those maintaining sinus rhythm. miR-208a was the most underexpressed [fold change (FC) = 2.458] and miR-483-5p the most overexpressed (FC = 1.804). miR-483-5p also demonstrated significant overexpression in the pre-operative serum of these patients, with ROC analysis demonstrating an overall predictive accuracy of 78%.

**CONCLUSIONS:**

This study provides the first description of atrial myocardial and circulating plasma microRNA in POAF patients. Our findings suggest POAF may be associated with pre-existing atrial substrate differences predisposing to arrhythmogenesis. Moreover, this study highlights the potential for miR-483-5p in biomarker development. Further work must now perform prospective, targeted validation of these results in a larger patient cohort.

## INTRODUCTION

*De novo* post-operative atrial fibrillation (POAF) affects ∼1 in 3 patients undergoing cardiac surgery leading to increased post-operative hospital stay, morbidity and mortality [1, 2]. Furthermore, POAF may also produce significantly worse long-term outcomes [3, 4], with evidence to suggest that 10-year mortality may be up to 48% higher in patients developing POAF when compared with those remaining in sinus rhythm (SR) [5].

POAF results from a complex interaction of ‘triggering’ stimuli and ‘sustaining’ mechanisms acting on a pro-arrhythmogenic myocardial substrate. While much investigation has focused on the stimulus for POAF, the make-up or indeed existence of any predisposing atrial substrate remains poorly understood.

First discovered in 1993 [6], microRNAs are small non-coding RNA molecules 19–25 nucleotides in length that suppress their target mRNA by inhibiting translation or promoting degradation. Although some are tissue specific, microRNAs are also present in the circulating blood, which derived from endothelial cells, platelets, mononuclear phagocytes or dead and dying cells. Circulating miRNAs are remarkably stable through carriage in apoptotic bodies and microvesicles, or binding to Argonaute 2 protein (Ago2) demonstrates resistance to endogenous RNases. As a result, they hold the potential to exert paracrine effects and even specifically deliver miRNA to their target cells [7]. However, the low concentration of paracellular miRNA detectable in human plasma and other biofluids as well as the common expression between blood cells and the organ/disease of interest has previously led to difficulty in interpreting the significance of circulating miRNA particularly in the context of biomarker discovery [8].

The role of miRNA in cardiac arrhythmogenesis is becoming increasingly apparent. miRNA targeting pathways associated with the regulation of cardiomyocyte metabolism (miR-208a and miR-223) may alter the provision of energy substrate required to maintain AF [9–11], whereas other miRNAs are thought to play a central role in changes associated with structural (miR-133, miR-590, miR-29b, miR-208, miR-638 and miR-150) and electrical remodelling (miR-328, miR-1 and miR-26) [12]. However, much of the work to date examines miRNA expression in right or left atrial tissue and there remains a paucity of evidence surrounding circulating microRNA in human AF [13, 14]. Furthermore, current studies address the circulating miRNA signature in long-standing and paroxysmal AF and do not examine the role of miRNA in the new onset post-operative form of this arrhythmia.

The purpose of this study is therefore to ascertain whether the atrial microRNA signature is altered in AF naive patients developing *de novo* post-operative AF. Furthermore, we aim to determine whether POAF is associated with any changes in circulating microRNA expression that may be detected pre-operatively, giving rise to the potential for biomarker development and a subsequent novel pre-operative risk stratification system in this highly prevalent and troublesome post-operative arrhythmia.

## METHODS

### Patient selection and recruitment

The study was approved by the local and regional research ethics committee (Ref: 09/H0711/23). Between November 2010 and September 2011, 34 patients undergoing non-emergent, on-pump coronary artery bypass grafting (CABG) at Imperial College Healthcare NHS Trust were prospectively selected to participate in this study and informed consent obtained. Emergency cases, those requiring adjunctive procedures (e.g. valve repair or replacement), patients with a prior history of any cardiac arrhythmia, thyroid disease, those taking anti-arrhythmic agents or undergoing surgery with mini-cardiopulmonary bypass systems were excluded.

All patients underwent continuous Holter (Novocor Vista 5 lead system, 2 channel recording) monitoring from the time of admission to the time of surgery (12–24 h). Post-operatively, all patients were monitored with the same Holter devices from the time of surgery to the time of discharge. Atrial fibrillation was defined according to Heart Rhythm Society Guidelines [15]. POAF was defined as new onset AF following CABG surgery in patients with pre-operative Holter recordings demonstrating SR and no prior history of the arrhythmia. All episodes of AF, atrial flutter or tachycardia of at least 30 s duration were documented. Only AF episodes >30 s were categorized as AF positive (Group 2) [15]. Patients were grouped retrospectively (following Holter analysis after discharge) according to the absence (Group 1) or presence (Group 2) of POAF.

### Sampling

Right atrial tissue biopsies were taken intra-operatively prior to instigation of cardiopulmonary bypass from the right atrial cannulation site. Whole blood samples were obtained at 24 h pre-operatively and at 2 and 4 days post-operatively. Plasma was extracted from whole blood by centrifugation at 5000 g for 6 min and aliquots stored at −80 °C until use.

### Laboratory methods

#### RNA extraction

Whole RNA was extracted from atrial tissue using TRIzol® reagent as described previously [16]. RNA quality and concentrations were assessed using the Nanodrop 1000 spectrophotometer and Agilent 2100 Bioanalyser. All RNA integrity numbers (RIN) were >7.5.

#### miRNA microarray

Microarray analysis was performed using the Agilent 60k microarray platform according to the manufacturer’s protocol [17]. Spike in solution derived from drosophila sequences was used to distinguish biological data from processing derived changes. One hundred nanaograms of total RNAs were ligated and fluorescently labelled with cyanine dye (pCp-Cy3) at its 3′ end. Labelled RNA was purified and dried prior to hybridization. Samples were loaded onto pre-prepared 8 × 60k MicroArray Slides (Agilent) and hybridized using the Agilent Sure-hyb hybridization chamber for 20 h at 55 °C. Following completion of hybridization, slides were scanned using the Agilent G2505C MicroArray Scanner and feature extraction performed using the Agilent Feature Extraction Software version 10.7.3.1. Analysis was performed using Agilent GeneSpring software GX11.0. A fold change (FC) cut-off of >1.5, at the *P* < 0.05 significance level, was set to detect miRNA differentially expressed between groups.

#### Quantitative reverse transcription polymerase chain reaction

miRNA changes were verified using specific TaqMan™ qPCR microRNA assays according to the manufacturer’s protocol (Taqman, Applied Biosystems, Life Technologies, Paisley, UK). For each study probe, identical quantitative reverse transcription polymerase chain reactions were also carried out using a control gene (miRNA-186), which was shown to be experimentally consistent across all samples in microarray analysis.

All reactions were carried out in three technical replicates using the Applied Biosystems 7500 fast Real-Time PCR System [18]. Amplification plots were examined for adequate amplification and successful polymerase chain reaction. A ΔR_n_ threshold value of 0.2 (within the exponential phase of amplification) was set to ensure comparability across plates. *C*_t_ values were recorded for both samples and controls and compared using the ΔΔ*C*_t_ method.

#### Statistical analysis

Inter-group comparisons were performed using Student's *t*-test if two groups or one-way analysis of variance if multiple groups. Statistical significance was reported if *P* < 0.05. Receiver operating characteristic (ROC) analysis was used to determine the diagnostic accuracy of pre-operative serum miRNA as a diagnostic biomarker for POAF. All calculations were performed using Prism (Graphpad, La Jolla, CA, USA).

## RESULTS

Thirty-four patients undergoing non-emergent, on-pump CABG were recruited. Twenty-one patients did not develop POAF and were classified into Group 1, and 13 patients developed POAF and were classified into Group 2. The mean time to onset of AF was 2.5 days.

### Pre-operative demographics

A summary of the pre-operative demographics is shown in Tables 1 and 2. No statistically significant differences were observed in any demographics or cardiovascular risk factors studied. Furthermore, no significant differences were seen between the groups in terms of β-blocker use or pre-operative echocardiographic parameters (Supplementary Table 1).

**Table 1:** Pre-operative demographics: cohort of patients in atrial tissue microarray experiments

	AF (*n* = 11)	nAF (*n* = 11)	*P*-value
Gender (% male)	73%	82%	0.193
Age (years)	63.3 ± 3.08	59.6 ± 3.62	0.452
BMI (kg/m^2^)	30.5 ± 2.29	27.1 ± 1.09	0.210
Height (m)	1.61 ± 0.07	1.72 ± 0.02	0.178
Weight (kg)	79.8 ± 4.31	81.3 ± 3.29	0.795
MI	27%	36%	0.647
Stroke	0.0%	18%	0.138
PVD	9.1%	9.1%	1.000
PCI	0.0%	0.0%	1.000
Hypertension	73%	73%	1.000
Hypercholesterolaemia	91%	91%	1.000
Family history	45%	36%	0.653
Diabetes	55%	55%	1.000
On insulin	27%	45%	1.000
Smoking	18%	27%	0.611
Alcohol >10 U per week	18%	36%	0.338

AF: atrial fibrillation; BMI: body mass index; MI: myocardial infarction; PVD: peripheral vascular disease; PCI: percutaneous coronary intervention.

**Table 2:** Pre-operative demographics: all patients in serum quantitative reverse transcription polymerase chain reaction experiments

	AF (*n* = 13)	nAF (*n* = 21)	*P*-value
Gender (M/F)	9/4	15/6	0.716
Age (years)	64.6 ± 11.3	59.6 ± 12.1	0.237
BMI (kg/m^2^)	30.2 ± 6.14	27.5 ± 3.41	0.247
Height (m)	1.62 ± 0.19	1.67 ± 0.08	0.475
Weight (kg)	80.1 ± 3.86	77.7 ± 2.69	0.623
MI	33%	24%	0.555
Stroke	8.3%	14%	0.614
PVD	17%	4.8%	0.252
PCI	0.0%	0.0%	1.000
Hypertension	75%	76%	0.939
Hypercholesterolaemia	92%	90%	0.909
Family history	45%	65%	0.291
Diabetes	58%	43%	0.392
On insulin	25%	24%	0.939
Smoking	17%	24%	0.629
Alcohol >10 U per week	25%	25%	1.000

AF: atrial fibrillation; BMI: body mass index; MI: myocardial infarction; PVD: peripheral vascular disease; PCI: percutaneous coronary intervention.

### miRNA microarray

A cohort of 22 matched samples (11 AF and 11 nAF) determined to have an RIN >7.5 were selected for miRNA microarray analysis. Based on a cut-off for significance at a FC of 1.5 and a *P*-value of <0.05, 16 miRNAs were differentially expressed between Groups 1 and 2. Of these, hsa-miR-483-5p was found to be the most up-regulated mature miRNA (FC = 1.804) and hsa-miR-208a the most down-regulated (FC = 2.458) (Table 3).

**Table 3:** Summary of microarray findings demonstrating 16 miRNAs differentially expressed between Group 1 (*n* = 11 SR) and Group 2 (*n* = 11 POAF) in atrial tissue

miRNA	*P*-value	Absolute fold change (FCa)	Regulation
hsa-miR-1202	0.0448	1.6767	Up
hsa-miR-150	0.0109	1.6919	Up
hsa-miR-19a	0.0400	1.6059	Down
hsa-miR-208a	0.0205	2.4580	Down
hsa-miR-223	0.0464	1.6014	Up
hsa-miR-24-1*	0.0134	1.8085	Down
hsa-miR-2861	0.0343	1.5234	Up
hsa-miR-29b	0.0298	1.7646	Down
hsa-miR-301a	0.0319	1.6051	Down
hsa-miR-3141	0.0178	1.5238	Up
hsa-miR-3679-5p	0.0328	1.7947	Up
hsa-miR-4298	0.0248	1.6312	Up
hsa-miR-483-5p	0.0193	1.8036	Up
hsa-miR-572	0.0197	1.6868	Up
hsa-miR-638	0.0353	1.5246	Up
kshv-miR-K12-3	0.0250	1.5513	Up

POAF: post-operative atrial fibrillation.

### Quantitative reverse transcription polymerase chain reaction

**Figure 1:**
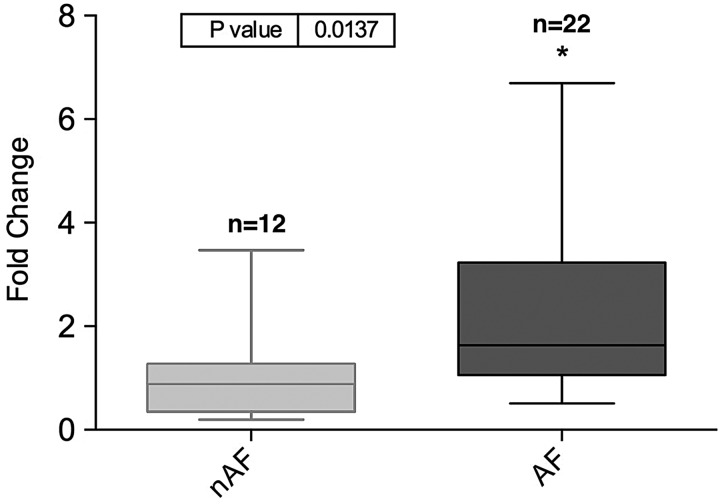
Expression of hsa-miR-483-5p in pre-operative serum (Group 1: *n* = 23; Group 2: *n* = 10).

**Figure 2:**
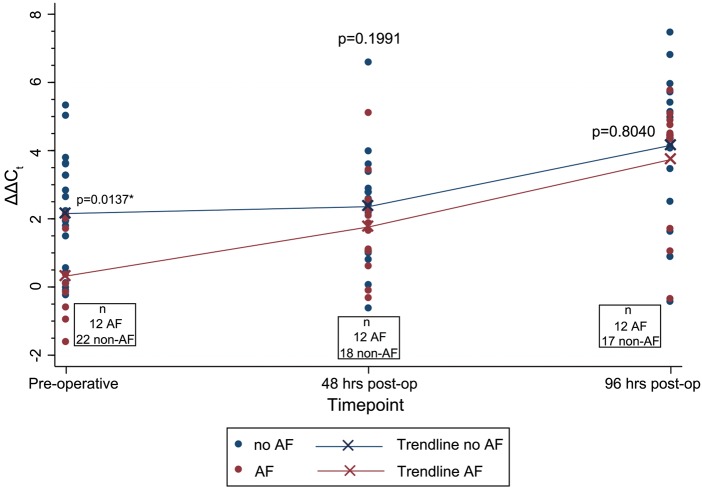
Scatter plot demonstrating expression levels of microRNA-483-5p at three different time points: pre-operatively, 48-h post-operatively and 96-h post-operatively. Large crosses and trend line demonstrate mean ΔΔ*C*_t_ values over time separated according to the presence or absence of POAF. *P*-values denote results of significance testing (Mann–Whitney) between AF and non-AF groups at each time point. AF: atrial fibrillation; POAF: post-operative atrial fibrillation.

In light of the differential expression in atrial tissue miRNA between groups, expressions of hsa-miR-483-5p and hsa-miR-208a were also examined in human serum at the following time points: (i) pre-operatively, (ii) 48 h post-operatively and (iii) 96 h post-operatively.

hsa-miR-208a is a cardiomyocyte-specific microRNA and was not detected in the serum at any time point (data not shown). hsa-miR-483-5p was found to be significantly higher in the pre-operative serum of Group 2 (POAF) when compared with Group 1 (SR) (*P* = 0.0137) (Fig. 1). No significant difference was observed in the serum expression between POAF and SR groups at 48-h (*P* = 0.1991) or at 96-h (*P* = 0.8040) time points (Fig. 2).

Figure 2 also demonstrates the change in miR-483-5p expression over time. There was a significant increase in the expression of miR-483-5p comparing pre-operative and 48-h time points in Group 2 (POAF) (*P* = 0.046); however, there was no significant change in expression between these time points in Group 1 (SR) (*P* = 0.9507). Both groups demonstrated a significant increase in miR-483-5p expression between 48 and 96 h post-operative time points (POAF: *P* = 0.0051; SR: *P* = 0.0055).

### Developing a serum test for post-operative atrial fibrillation

**Figure 3:**
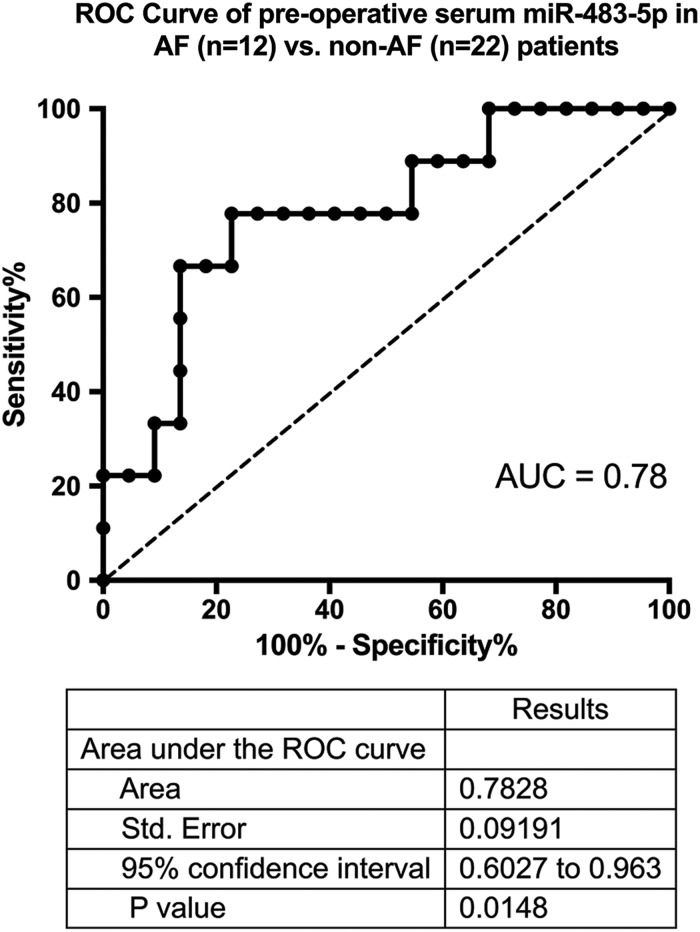
Receiver operating characteristic (ROC) curve of pre-operative hsa-miR-483-5p as a biomarker for post-operative atrial fibrillation (AF).

ROC analysis revealed hsa-miR-483-5p levels to have a sensitivity and specificity of 77.78 and 77.27, respectively, with a likelihood ratio of 3.422 for the prediction of post-operative AF when a FC cut-off of 1.262 was used for the expression level of miRNA-483-5p. The area under the curve (AUC) was 0.78 (Fig. 3).

## DISCUSSION

The potential for microRNA to act as non-invasive molecular biomarkers of systemic disease is of great interest owing to their high level of stability in many different biofluids including serum, plasma and urine [19]. As such, by means of a simple blood test performed at the time of routine patient assessment, quantitation of a single or cluster of microRNAs may provide a novel avenue for detection and risk prediction of systemic disease with minimal disturbance to the patient.

The underlying pathophysiology of atrial fibrillation is complex, and a combination of structural myocardial changes, alterations in the extracellular environment, intracellular ion flux and systemic circulatory factors is likely to underlie the pro-arrhythmogenic substrate [20]. Post-transcriptional regulation of target genes by miRNA may provide one mechanism by which these processes may be regulated, disrupting cellular homeostasis and precipitating arrhythmia. Indeed, recently Liu *et al*. identified circulating microRNA-150 to be significantly underexpressed in the serum of 60 prospectively collected patients with both paroxysmal (*n* = 30; FC ∼ 17) and persistent (*n* = 30; FC ∼ 20) AF when compared with healthy controls (*n* = 30), demonstrating miR-150 to be an independent predictor of AF (OR: 1.96; 95% CI: 1.5, 3.57; *P*< 0.001). However, miRNA-150 levels were not able to differentiate paroxysmal from persistent AF (OR: 1.02; 95% CI: 0.82, 1.13; *P* = 0.61) [13]. Dawson *et al*. also demonstrated a significant down-regulation of miR-29b in both the plasma (*n* = 30SR, 33 AF) and right atrial appendage tissue of patients with paroxysmal (*n* = 9) and chronic atrial fibrillation (*n* = 8) with a subsequent reduction in suppression of fibrilin and the collagen (COL1A1 and COL3A1) mRNA transcripts promoting atrial fibrosis, structural remodelling and increasing the susceptibility to re-entry and atrial fibrillation [14].

This study for the first time examines the role of miRNA in the circulating plasma of POAF and our results provide the first documented insights into the microRNA signature of this condition. Sixteen microRNA exhibit significant differential expression in the atria of POAF patients, of which miR-208a is the most down-regulated and miR-483-5p the most up-regulated mature microRNA. miR-483-5p is also expressed at significantly higher levels in the pre-operative serum of AF naive patients going on to develop *de novo* AF in the post-operative period. The expression of this miRNA then normalizes to that of patients without dysrhythmia by 48 h after surgery. Furthermore, at a cut-off FC of 1.26, ROC analysis reveals a diagnostic accuracy of 78% (AUC: 0.78) when this biomarker is used to predict patients developing POAF. This compares favourably with previously described pre-operative biomarkers such as pro-BNP, C-reactive protein and peripheral blood white cell count, which have been reported to have diagnostic accuracies of 61, 68 and 69%, respectively, when predicting POAF [21–23].

MicroRNA 483-5p is a 22-nucleotide (AAGACGGGAGGAAAGAAGGGAG) intronic mature microRNA typically transcribed with its host gene, *IGF2*, located on chromosome 11p15.5 [24]. It has been isolated in several human tissue types including myocardium, hepatic and brain tissue as well as in blood serum [24, 25]. At present, miR-483-5p remains relatively poorly examined and there are no studies reporting on the use of circulating miR-483-5p as a serum biomarker. Furthermore, the mechanism by which this microRNA regulates or is modulated by the clinical phenotype of POAF remains undetermined. Recent work by Morley-Smith *et al*. [26] has demonstrated an acute and sustained increase in circulating mir-483-3p (which originates from the opposite arm of the same pre-miRNA as miR-483-5p) in heart failure patients after left ventricular assist device insertion, mirroring suppression of NT-pro-BNP secretion. Alongside our results, these findings may suggest that transcription of serum pre-miRNA-483 may be stimulated in states of cardiac stress, the mature products of which may then be detected in the systemic circulation. It should also be considered however that increased miR-483 may represent a bystander effect rather than a direct miRNA-mediated mechanism of POAF pathogenesis. Indeed, the low concentration of paracellular miRNA detectable in human plasma argues against a substantial paracrine function particularly given the one-to-one nature of microRNA–mRNA interaction that typically requires high levels of intracellular microRNA [27]. It is therefore possible that the observed increase in miR-483-5p expression represents up-regulation of its host gene *IGF2*, which, for example, may be stimulated in an attempt to drive cardiac regeneration in states of cardiac stress. Overexpression of *IGF2* may also play a role in providing a pro-inflammatory substrate through the regulation of NF-kB- and IL-6-mediated pathways, which may in turn lower the threshold on which a surgical trigger may potentiate AF in the post-operative period [28]. As such, the exact mechanistic role of miR-483-5p beyond its use as a simple biomarker requires further investigation with examination not only of this miRNA but also of its host gene transcription and protein expression.

### Limitations

It is important to consider a number of limitations when interpreting these results. First, although the rigorous nature of this trial provides a robust definition of POAF with prospectively collected data, the numbers of patients recruited remain small and further validation is required in a larger patient cohort. Second, continuous post-operative Holter monitoring for AF was limited to the in-hospital period and as such asymptomatic AF occurring beyond this time point may have remained undetected. Third, AF is a complex, multifactorial arrhythmia and microRNA dysregulation alone is unlikely to be the sole regulatory mechanism underlying its pathogenesis. Furthermore, although every attempt was made to match for environmental disparities, confounding factors such as electrolyte imbalance, post-operative sepsis (clinical or sub-clinical) and pharmacological therapies may all influence the relative expression of microRNA and the regulation of subsequent biological pathways. Finally, common miRNA expression between blood cells and the organ/disease of interest may also lead to difficulty in interpreting the significance of circulating miRNA particularly in the context of biomarker discovery [8].

## CONCLUSIONS

This work has identified potential pre-existing microRNA dysregulation in patients subsequently developing POAF, particularly demonstrating underexpression of miR-208a and overexpression of miR-483-5p in the right atrium of POAF patients. In order to understand the significance of these changes, it is now pertinent to clarify the potential mechanisms by which these miRNA may regulate cardiac conduction.

In addition, this study has provided the first documented description of circulating microRNA in POAF patients. Indeed, the observed differential expression of miRNA-483-5p in both the right atrial tissue and circulating plasma of these patients supports the hypothesis of a pre-existing atrial or systemic inflammatory substrate to this condition, highlighting the potential for biomarker development. Given these findings, it is now necessary to address the potential mechanisms behind these changes and validate these findings. Prospective, targeted validation of these results in a larger patient cohort is now required in order to determine the reliability of circulating microRNA-483-5p as a biomarker for POAF.

## SUPPLEMENTARY MATERIAL

Supplementary material is available at *EJCTS* online.

## Funding

We are grateful to the Wellcome Trust (Ref: WT100023MA) and Imperial College Charity (Ref: 5117/R20R) for providing the financial support that made this work possible. Funding to pay the Open Access publication charges for this article was provided by the Imperial College London Charity Open Access Fund (COAF).

**Conflict of interest:** none declared.

## Supplementary Material

Supplementary Data
