# Supplementary material for: Elevated serum microRNA 483-5p levels may predict patients at risk of post-operative atrial fibrillation
Source: Eur J Cardiothorac Surg. 2016 Jul 15;51(1):73–8. doi: 10.1093/ejcts/ezw245 (PMC5226070; doi:10.1093/ejcts/ezw245)
Supplement: Supplementary Data [file supp_51_1_73__index.html]

Elevated serum microRNA 483-5p levels may predict patients at risk of post-operative atrial fibrillation — Supplementary Data 

# Elevated serum microRNA 483-5p levels may predict patients at risk of post-operative atrial fibrillation

## Supplementary Data

Supplementary Data

- Supplementary Table 1 - docx file
