# Supplementary material for: Elevated serum microRNA 483-5p levels may predict patients at risk of post-operative atrial fibrillation
Source: Eur J Cardiothorac Surg. 2016 Jul 15;51(1):73–8. doi: 10.1093/ejcts/ezw245 (PMC5226070; doi:10.1093/ejcts/ezw245)
Supplement: Supplementary Data [file supp_ezw245_ezw245supp_table1.docx]

**Supplementary Table 1.** Pre-operative Echocardiographic parameters

|  |  | ***Mean ± SD*** | | |
| --- | --- | --- | --- | --- |
|  | ***n*** | ***AF*** | ***non-AF*** | ***p*** |
| LA systolic diameter (cm) | 9 AF, 12 nAF | 3.83±0.91 | 3.90±3.54 | 0.8504 |
| Dilated RA [%(n)] | 9 AF, 13 nAF | 22.2% (2) | 15.4% (2) | 0.683 |
| LV Diastolic Diameter (cm) | 9 AF, 14 nAF | 4.72±1.06 | 4.59±0.51 | 0.7305 |
| LV Systolic Diameter (cm) | 9 AF, 13 nAF | 3.17±0.94 | 3.17±0.38 | 0.9964 |
| Fractional Shortening (%) | 8 AF, 11 nAF | 34.1±10.4 | 33.9±2.34 | 0.8998 |
| IVS diastolic thickness (cm) | 9 AF, 13 nAF | 1.00±0.18 | 1.05±0.14 | 0.5756 |
| LVPW diastolic thickness (cm) | 9 AF, 12 nAF | 0.90±0.17 | 1.02±0.16 | 0.1911 |
| Aortic Root diameter (cm) | 9 AF, 12 nAF | 3.19±0.32 | 3.18±0.60 | 0.9528 |
| AV peak gradient (mmHg) | 9 AF, 14 nAF | 9.72±6.67 | 7.25±3.26 | 0.3239 |
| LVOT peak velocity (cm/s) | 9 AF, 10 nAF | 89.6±19.4 | 96.2±21.3 | 0.4847 |
| LVOT peak gradient (mmHg) | 9 AF, 10 nAF | 4.37±3.56 | 3.84±1.57 | 0.6898 |
| MV E'/A' ratio | 8 AF, 14 nAF | 0.83±0.27 | 0.85±0.24 | 0.9055 |

**Legend:** AF– Atrial fibrillation; DM– Diabetes Mellitus; BMI– Body Mass Index; FH– Family History; CHOL– Hypercholesterolaemia; HBP– hypertension; PCI– Previous percutaneous coronary intervention; PVD– Peripheral Vascular Disease; CVA– Stroke; TIA– Transient Ischaemic Attack; MI– Myocardial Infarction
